# Reliability of the Cerebral Performance Category to classify neurological status among survivors of ventricular fibrillation arrest: a cohort study

**DOI:** 10.1186/1757-7241-19-38

**Published:** 2011-06-15

**Authors:** Kamal Ajam, Laura S Gold, Stacey S Beck, Susan Damon, Randi Phelps, Thomas D Rea

**Affiliations:** 1 The Department of Medicine, University of Washington, (NE Pacific Street) Seattle 98195, USA; 2 The Division of Emergency Medical Services, Public Health - Seattle & King County, (401 5th Ave) Seattle 98104, USA

**Keywords:** Ventricular fibrillation, heart arrest, neurological status, Cerebral Performance Category

## Abstract

**Background:**

The Cerebral Performance Category (CPC) score is widely used in research and quality assurance to assess neurologic outcome following cardiac arrest. However, little is known about the inter- and intra-reviewer reliability of the CPC.

**Methods:**

We undertook an investigation to assess the inter-reviewer and source document reliability of the CPC among a cohort of survivors from out-of-hospital ventricular fibrillation cardiac arrest (n = 131) in a large metropolitan area between November 1, 2003 and December 31, 2005. Subjects with a CPC of 1 or 2 were classified as favorable outcome and those with CPC 3 or greater were classified as unfavorable outcome. One abstractor first used the discharge summary alone to determine the CPC. All 3 abstractors independently reviewed the entire hospital record. Reliability was assessed by determining the proportion of determinations that agreed between abstractors and the respective kappa statistics. We also evaluated the implications for determining survival with favorable neurological outcome when survival to hospital discharge was 20% and 30%.

**Results:**

When the entire hospital record was used to determine CPC, favorable neurologic outcome (CPC 1 or 2) was recorded in 92% by abstractor 1, 89% by abstractor 2, and 74% by abstractor 3. Agreement was 96% (kappa = 0.78) between abstractors 1 and 2, 84% (kappa = 0.49) between abstractors 2 and 3, 82% (kappa = 0.38) between abstractors 1 and 3. The 3-way kappa was 0.50. Agreement was 90% (kappa = 0.71) between the discharge summary alone and the entire hospital record. If the results from review of the entire record are applied to a circumstance where survival to discharge is 20%, favorable neurologic status would occur in 18.4% for abstractor 1, 17.8% for abstractor 2, and 14.8% for abstractor 3. For survival to hospital discharge of 30%, favorable neurologic status would occur in 27.6% for abstractor 1, 26.7% for abstractor 2, and 22.2% for abstractor 3.

**Conclusions:**

In this cohort study of survivors of out-of-hospital ventricular fibrillation cardiac arrest, the use of the CPC to classify favorable versus unfavorable neurological status at hospital discharge produced variable inter- and intra-reviewer agreement. The findings provide useful context to interpret outcome evaluations that report CPC.

## Introduction

Optimal survival following sudden cardiac arrest requires heart and brain resuscitation. In patients who achieve cardiac resuscitation, brain recovery from anoxic injury is variable. Neurological sequelae range from complete recovery to coma with brain death. (1,2) Thus, ideally outcome assessment would incorporate functional and neurologic status.

Several assessment tools are available; however the Cerebral Performance Category (CPC) score is widely used in research and quality assurance. (3-5) Evidence suggests that the CPC corresponds - though imperfectly - to quality of life and functional status derived from more extensive evaluation. (6,7) Although the CPC is often used to assess outcome, little is known about its methodological characteristics. A high degree of intra- and inter-reviewer agreement would help enable valid and robust comparison of neurologic outcome. In contrast, poor agreement could undermine the usefulness of evaluating neurologic outcome and detract from the robustness of studies that compare neurologic outcomes. A better understanding of the CPC measurement characteristics could aid study design and interpretation and help inform the potential limitations and biases of neurologic recovery following cardiac arrest. We undertook a an investigation to assess the inter-reviewer and source document reliability of the CPC at hospital discharge among a cohort of survivors from out-of-hospital ventricular fibrillation cardiac arrest.

## Methods

### Study design, setting and population

The investigation was a cohort medical record study of patients who were resuscitated from out-of-hospital ventricular fibrillation arrest due to heart disease and discharged alive from the hospital in Seattle and King County from November 1, 2003 through December 31, 2005. King County including Seattle has an area of approximately 2000 square miles, a population of 1.75 million, and includes urban, suburban, and rural areas. The area is served by a two-tiered emergency medical services (EMS) system that is activated by calling 9-1-1 and speaking with an emergency dispatcher. The first tier consists of fire fighter-emergency medical technicians who are trained in basic life support and automated defibrillation. The second tier consists of paramedics who are trained in advanced life support. There are 12 hospitals covering this service area. The appropriate Institutional Review Boards approved the study methods. The authors had full access to the data and take responsibility for its integrity.

### Data Collection and Definitions

The Seattle Fire Department and King County EMS maintain a surveillance system in order to review EMS care and outcome of out-of-hospital cardiac arrest. (8) Persons who suffered out-of-hospital ventricular fibrillation and survived to be discharged alive from the hospital were invited approximately 6 months after discharge to participate in an investigation of care and outcomes of cardiac arrest. For those who provided written consent, we obtained a copy of their entire hospital medical records for the stay related to the arrest.

The CPC ranges from 1 to 5 with 1 representing intact function and 5 representing brain death (Table 1) (4). Many researchers and quality assurance personnel classify favorable neurological function as CPC 1 or 2 and unfavorable function as 3 or greater (3,5). Consequently - though abstractors classified subjects on the 1 to 5 scale, we classified subjects into 2 nominal groups for the purposes of current study. Subjects with a CPC of 1 or 2 were classified as favorable outcome and subjects with a CPC of 3 or greater were classified as unfavorable outcome.

**Table 1:** Cerebral Performance Category

**1**. **Good Cerebral Performance **(*Normal Life*)	Conscious, alert, able to work and lead a normal life. May have minor psychological or neurologic deficits (mild dysphasia, nonincapacitating hemiparesis, or minor cranial nerve abnormalities).
**2**. **Moderate Cerebral Disability **(*Disabled but Independent*)	Conscious. Sufficient cerebral function for part-time work in sheltered environment or independent activities of daily life (dress, travel by public transportation, food preparation). May have hemiplegia, seizures, ataxia, dysarthria, dysphasia, or permanent memory or mental changes.

**3. Severe Cerebral Disability **(*Conscious but Disabled and Dependent*)	Conscious; dependent on others for daily support (in an institution or at home with exceptional family effort). Has at least limited cognition. This category includes a wide range of cerebral abnormalities, from patients who are ambulatory but have severe memory disturbances or dementia precluding independent existence to those who are paralyzed and can communicate only with their eyes, as in the locked-in syndrome.

**4. Coma/Vegetative State **(*Unconscious*)	Unconscious, unaware of surroundings, no cognition. No verbal or psychologic interaction with environment.

**5. Brain Death **(*Certified brain dead or dead by traditional criteria*)	Certified brain dead or dead by traditional criteria.

The abstractors were asked to determine the CPC at the time of hospital discharge through hospital record review that involved only the specific hospitalization related to the resuscitation. Each abstractor was not aware of fellow abstractors CPC ratings and so independently completed reviews to determine CPC. The first 2 abstractors used the entire hospital record including notes from physicians, nursing, and ancillary professionals as well information from diagnostic, imaging, and laboratory tests. The third abstractor conducted 2 separate reviews. This abstractor first used only the discharge summary to determine the CPC score. After an interval of approximately 3 months, records were then randomly reordered, and the same abstractor used the entire hospital record to determine the CPC. The review process occurred over several months. The reviewers all had medical backgrounds: one was a clinically-active, hospital-based physician with training in internal medicine, one was a clinical research nurse with substantial experience in cardiac arrest research including CPR training and medical record review, and one was a senior medical student who had completed her core clinical training. The abstractors had the CPC description available for reference during the review but did not have other special guidance or opportunity for consensus review.

### Data Analysis

We constructed 2 × 2 tables comparing the CPC between the abstractors based on the entire hospital record review. We constructed a 2 × 2 table comparing the CPC between the discharge summary and the entire hospital record for the single abstractor. We report the percentage of agreement from the 2 × 2 tables. We also calculated the unweighted 2-way and when appropriate 3-way kappa coefficients for the comparisons. We used the results to assess the potential differences in favorable neurologic outcome when survival to hospital discharge was set at 20% and 30%. We chose these survival measures as they approximate global summary estimates (20%) and the historical survival for the study community (30%) (9, 10). Analyses were conducted using Stata 8.0.

## Results

During the 26 months of study, 231 persons suffered out-of-hospital ventricular fibrillation cardiac arrest due to heart disease and were resuscitated and discharged alive from the hospital. Of these 231, 9 had died by the time of potential study contact, 5 had a language barrier, 30 could not be contacted, 29 verbally agreed but did not sign a consent form, 20 declined participation, and 7 did not have their full record available or were not reviewed by all 3 abstractors, leaving a total study population of 131. When the entire hospital record was used to determine CPC, favorable neurologic outcome (CPC 1 or 2) was recorded in 92% by abstractor 1, 89% by abstractor 2, and 74% by abstractor 3. A favorable CPC was recorded in 84% by abstractor 3 when only the discharge summary was used to determine the CPC.

When the entire hospital record was used to determine CPC, agreement was 96% (kappa = 0.78) between abstractors 1 and 2, 84% (kappa = 0.49) between abstractors 2 and 3, 82% (kappa = 0.38) between abstractors 1 and 3 (Tables 2, 3, 4). The 3-way kappa score among all abstractors when the entire hospital chart was used was 0.50. As illustrated by Tables 2, 3, and 4, the disagreement was predominantly unidirectional. Specifically one reviewer consistently coded unfavorable CPC while the other reviewers coded favorable CPC, as opposed to the favorable-unfavorable disagreement being equally distributed between the abstractors. Agreement was 90% (kappa = 0.71) between the discharge summary alone and the entire hospital record (Table 5).

**Table 2:** CPC scores of abstractors 1 and 2 using complete hospital charts

		Abstractor 2 (complete hospital chart)		Total
			
		Favorable CPC (1, 2)	Unfavorable CPC (3, 4)	
**Abstractor 1 (complete hospital chart)**	Favorable CPC (1, 2)	116	5	121
	
	Unfavorable CPC (3, 4)	0	10	10

**Total**		116	15	131

**Table 3:** CPC scores of abstractors 2 and 3 using complete hospital charts

		Abstractor 3 (complete hospital chart)		Total
			
		Favorable CPC (1, 2)	Unfavorable CPC (3, 4)	
**Abstractor 2 (complete hospital chart)**	Favorable CPC (1, 2)	96	20	116
	
	Unfavorable CPC (3, 4)	1	14	15

**Total**		97	34	131

**Table 4:** CPC scores of abstractors 1 and 3 using complete hospital charts

		Abstractor 3 (complete hospital chart)		Total
			
		Favorable CPC (1, 2)	Unfavorable CPC (3, 4)	
**Abstractor 1 (complete hospital chart)**	Favorable CPC (1, 2)	97	24	121
	
	Unfavorable CPC (3, 4)	0	10	10

**Total**		97	34	131

**Table 5:** CPC scores of abstractor 3, using complete hospital charts and discharge summaries

		Abstractor 3 (complete hospital chart)		Total
			
		Favorable CPC (1, 2)	Unfavorable CPC (3, 4)	
**Abstractor 3 (discharge summary)**	Favorable CPC (1, 2)	97	13	110
	
	Unfavorable CPC (3, 4)	0	21	21

**Total**		97	34	131

If the results derived from review of the entire hospital record are applied to a circumstance where survival to hospital discharge is 20%, favorable neurologic status would occur in 18.4% for abstractor 1, 17.8% for abstractor 2, and 14.8% for abstractor 3. If the results derived from review of the entire hospital record are applied to a circumstance where survival to hospital discharge is 30%, favorable neurologic status would occur in 27.6% for abstractor 1, 26.7% for abstractor 2, and 22.2% for abstractor 3.

## Discussion

In this chart review study of survivors of out-of-hospital ventricular fibrillation cardiac arrest, the use of the CPC to classify favorable versus unfavorable neurological status at hospital discharge produced variable inter- and intra-reviewer agreement. Agreement ranged from 82% to 96% (kappa 0.38 to 0.78) with disagreement between abstractors being largely uni-directional. The CPC determined from just the discharge summary was more often favorable than the CPC determined from the entire hospital record. The level of (dis)agreement between abstractors observed in this study would produce a range in the proportion coded with favorable neurologic outcome of 22% to 28% if survival to hospital discharge was 30% and 15% to 18% if survival to hospital discharge was 20%

Functional and neurologic status following cardiac arrest is a more meaningful clinical outcome than simply hospital survival when trying to judge the effectiveness of resuscitation care. (11) Indeed newer therapies such as hypothermia are directed toward brain protection and recovery. Functional neurologic status consists of multiple domains including activities of daily living, cognitive function such as memory and abstract thought, and emotional health; domains that appear to change over the months after the arrest. Ideally then functional and neurologic status would derive from standard, validated, and repeated measures that involve direct subject communication and/or examination. In many circumstances however, the ability to undertake this type of evaluation is not feasible because of limited resources or the practical logistics of subject contact and participation. The CPC overcomes some of these challenges because assessment does not require direct subject contact and does not require assessment at specified time points (i.e. 3 or 6 months following resuscitation).

Although the CPC has some important and practical advantages, the current study enables a better understanding of its potential limitations and the implications for assessing outcome. The disagreement in coding could produce some bias. Randomized trials need to consider whether abstractors are evenly distributed across the randomization assignment. Otherwise the differential inter-reviewer variability could be a potential source for bias when interpreting the effectiveness of the study intervention. Similarly, evaluations of temporal trends including before and after studies should be aware of potential bias if abstractors change over time. Finally, these findings should be considered when comparing resuscitation effectiveness across EMS systems or communities.

Given the advantages and limitations of the CPC, certain approaches may attenuate the potential for bias. As per the Utstein template, the report of survival in conjunction with neurological status helps one interpret the findings. For example, one might expect that better neurologic survival would correspond to better overall survival. If not, then one must consider whether there is a clinical explanation for why changes in favorable neurologic status would not track with survival. Anecdotally, reviewers were able to determine the CPC with greater ease and certainty when reports from ancillary services - physical, occupational, and speech therapy - were available, as these reports provided specific detail regarding activities of daily living. As with stroke, cardiac arrest recovery may be optimized with a multidisciplinary approach that includes rehabilitation services. A secondary benefit of this multidisciplinary approach might be a more consistent CPC assessment.

This study has limitations. The study collapsed the CPC levels into 2 groups as is often reported in clinical and research studies. Although this approach was planned a-priori and selected to provide the most direct relevance to published practice, the study does not in the strictest sense report the reliability of each level of the CPC scale. Ideally the study would have many more reviewers to assess inter-reviewer agreement. However the findings resulted from the efforts of 3 reviewers, each with distinct backgrounds but all with clinical knowledge and experience. There was no opportunity for consensus or a specific training set. These differences may account for some of the variability. The intra-rater comparison did not use the same source documentation but rather initially used the discharge summary and then later the full chart. This strategy was chosen to determine if the discharge summary alone provided comparable assessment to the entire chart. Thus the comparison is not a pure intra-rater reliability test because of the difference in source documentation. Other approaches that enable consensus or provide additional description or reference examples may produce greater agreement. However, there is typically no standard training approach or reviewer experience requirement employed in clinical trials or programmatic assessment; so that the findings are likely consistent with current practice (12-14).

In addition, the study required written informed consent obtained typically 6 months after hospital discharge. As a consequence of this structure and requirement, a fair portion of the eligible cohort could not be assessed. The study occurred in a large regional EMS system where resuscitated patients receive care at one of 12 community or academic hospitals. Taken together, these characteristics could influence the generalizability of the results. For example, those who could not be contacted or who decline participation may have poorer neurological and functional status. We believe however that the findings are likely representative of the CPC characteristics. Although the current study helps define the inter-reviewer reliability of the CPC, the study did not evaluate what reviewer or hospital-record determinants are important for achieving a high level of consistency.

## Conclusion

In this cohort investigation of survivors of out-of-hospital ventricular fibrillation cardiac arrest, the use of the CPC to classify favorable versus unfavorable neurological status at hospital discharge produced variable inter- and intra-reviewer agreement. The findings provide useful context to interpret outcome evaluations that report the CPC. The CPC offers a relatively efficient approach to assess cardiac arrest outcomes. Going forward, approaches that provide more systematic chart-based information or provide more explicit guidance for reviewers may help maximize the clinical usefulness and reliability of the CPC.

## Abbreviations

CPC: Cerebral Performance Category

## Competing interests

The authors declare that they have no competing interests.

## Authors' contributions

KA acquired data and drafted the manuscript. LG performed data analysis and made critical revisions to the manuscript. SB acquired data and made critical revisions to the manuscript. SD acquired data and made critical revisions to the manuscript. RP managed the data, performed data analysis, and made critical revisions to the manuscript. TR conceived the research, acquired research support, and made critical revisions to the manuscript. Each author has read and approved the final manuscript.
